# The involvement of α-synucleinopathy in the disruption of microglial homeostasis contributes to the pathogenesis of Parkinson’s disease

**DOI:** 10.1186/s12964-023-01402-y

**Published:** 2024-01-12

**Authors:** Yongzhen Miao, Hongrui Meng

**Affiliations:** 1 Institute of Neuroscience, Soochow University, Suzhou, Jiangsu China; 2 Department of Neurology and Clinical Research Center of Neurological Disease, The Second Affiliated Hospital of Soochow University, Suzhou, Jiangsu China

**Keywords:** α-synuclein, Microglia, Parkinson's disease, Inflammation, Central nervous system

## Abstract

The intracellular deposition and intercellular transmission of α-synuclein (α-syn) are shared pathological characteristics among neurodegenerative disorders collectively known as α-synucleinopathies, including Parkinson's disease (PD). Although the precise triggers of α-synucleinopathies remain unclear, recent findings indicate that disruption of microglial homeostasis contributes to the pathogenesis of PD. Microglia play a crucial role in maintaining optimal neuronal function by ensuring a homeostatic environment, but this function is disrupted during the progression of α-syn pathology. The involvement of microglia in the accumulation, uptake, and clearance of aggregated proteins is critical for managing disease spread and progression caused by α-syn pathology. This review summarizes current knowledge on the interrelationships between microglia and α-synucleinopathies, focusing on the remarkable ability of microglia to recognize and internalize extracellular α-syn through diverse pathways. Microglia process α-syn intracellularly and intercellularly to facilitate the α-syn neuronal aggregation and cell-to-cell propagation. The conformational state of α-synuclein distinctly influences microglial inflammation, which can affect peripheral immune cells such as macrophages and lymphocytes and may regulate the pathogenesis of α-synucleinopathies. We also discuss ongoing research efforts to identify potential therapeutic approaches targeting both α-syn accumulation and inflammation in PD.

Video Abstract

**Supplementary Information:**

The online version contains supplementary material available at 10.1186/s12964-023-01402-y.

## Introduction

Parkinson's disease (PD) is a neurodegenerative disorder characterized by selective vulnerability of dopaminergic neurons in the midbrain substantia nigra pars compacta (SNpc) [1]. Degeneration of dopaminergic neurons in the SNpc that project to the striatum reduces dopamine input to the basal ganglia, resulting in motor symptoms such as bradykinesia and rigidity [2]. Another neuropathological feature of PD is the presence of Lewy bodies (LBs) and Lewy neurites (LNs), which are formed by abnormal deposition of α-synuclein (α-syn), dysmorphic organelles, vesicular structures, and lipids in the cytoplasm of neurons in several different brain regions [3, 4]. Although PD was initially described as a movement disorder closely related to dopaminergic neuron degeneration, other cell types throughout the central and peripheral autonomic nervous system are also involved. This may lead to various symptoms, including nonmotor hyposmia, sleep disorders, constipation, and cognitive and psychiatric symptoms such as dementia and depression [5].

Although most PD cases are sporadic, a substantial proportion of PD cases are attributed to variants or alterations in specific monogenic genes. To date, mutations in at least 20 genes have been identified to be responsible for PD [6]. Additionally, variants of these genes collectively play a role in the development of sporadic PD [7]. Intraneuronal protein aggregates consisting primarily of α-syn are found in most patients with PD. According to the findings of a large-scale genome-wide association study (GWAS), polymorphisms in noncoding regions of the α-syn gene, *SNCA,* are significant risk factors for idiopathic PD [8]. α-Syn can deviate from its native state, forming amyloid fibrils, ultimately resulting in the formation of cytoplasmic inclusions, which are associated with several neurodegenerative diseases, including PD [9, 10], dementia with Lewy bodies (DLB) [11, 12], and multiple system atrophy (MSA) [13, 14]. Various pathological α-syn aggregates can form from the same precursor protein, for instance, LBs in neurons [15] and GCIs (glial cytoplasmic inclusions) in glial cells [16], leading to the diverse clinical and pathological characteristics of α-synucleinopathies [17].

Microglia are the primary type of innate immune cells in the central nervous system (CNS), accounting for approximately 5–10% of all glial cells in the healthy human brain [18]. Mammalian microglia originate from myeloid precursor cells in yolk sac tissue before migrating to and colonizing the brain. In the CNS, microglia are considered to reach a stable state and are responsible for maintaining immune homeostasis and protecting the brain against diseases and pathogens [19]. α-Syn aggregation has been demonstrated to not only evoke the innate immune response but also recruit and activate the adaptive arms of the immune system in PD to promote neuroinflammation [20, 21], and microglial activation is the initial step in this process [22]. Additionally, microglial activation induced by inflammagen such as lipopolysaccharide (LPS), stimulates the aggregation of insoluble α-syn and exacerbates neuroinflammation [23, 24]. This implies that microglial activation and inflammation engage in a self-perpetuating cycle. Similar to macrophages, microglia play a crucial role in eliminating invading pathogenic bacteria, cell debris, and abnormal proteins from the CNS [25, 26]. Investigations involving positron emission tomography (PET) have revealed that microglia are activated in the brains of individuals with PD [27]. Recently, emerging evidence has suggested that microglia perform a variety of distinct roles, exhibiting a spectrum of phenotypes in the pathogenesis of PD. For example, under inflammatory conditions, microglia can secrete cytotoxic proinflammatory cytokines and directly promote dopaminergic neuron death; alternatively, activated microglia can scavenge debris and toxic metabolites from damaged neurons and other cells. Activated microglia can clear pathological α-syn through phagocytosis, and microglia activated by IL-6 may attenuate the number of α-syn inclusions in animal models [28]. Microglial phagocytosis can be selectively activated by the monomeric form of α-syn but is suppressed by aggregated α-syn and promotes α-syn-induced dopaminergic neurotoxicity, suggesting that microglia contribute to the pathological process of PD [29, 30]. In addition, several PD-related genes, such as *leucine-rich repeat kinase 2* (*LRRK2*) and *DJ-1*, are expressed in microglia and regulate microglial clearance [31–33], suggesting that microglial phagocytosis contributes to the development and progression of PD pathology. These findings demonstrate that phagocytosis of α-syn cooperates with intracellular events involved in α-syn processing by microglia, which is involved in neuronal deposition, spreading, and disease progression. Therefore, elucidating the association between microglial activation and α-syn aggregation and propagation may provide insight into the pathological progression of PD, which is critical for developing future therapies.

In this review, we summarize recent findings related to microglial activation and microglia-associated neuroinflammation that are relevant to α-syn aggregation and propagation. We focus our discussions on research developments related to cellular processes associated with inflammation, describing the current understanding of the connection between α-syn and the disruption of microglial homeostasis in the pathogenesis of PD. In addition, we also discuss current therapeutic approaches targeting microglia-mediated inflammation for preventing disease progression from the perspective of these new findings.

## Structure and conformation of α-syn

**Fig. 1:**
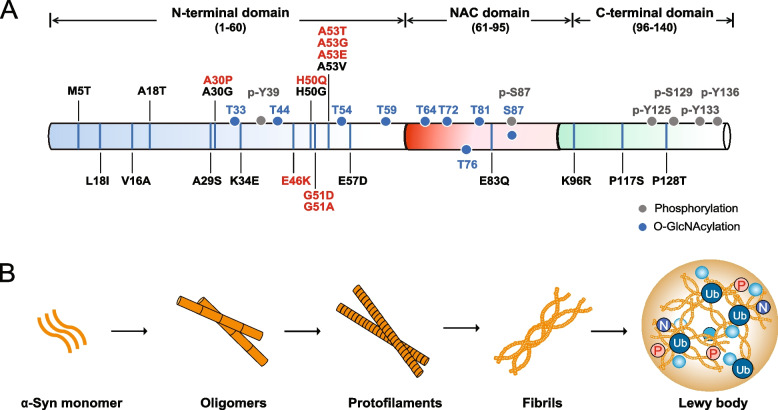
**A** Schematic of the domain structures, genetic mutations, and posttranscriptional modifications of α-syn related to Parkinson’s disease (PD). The α-synuclein (α-syn) protein comprises three domains: the N-terminal amphipathic region, the nonamyloidogenic component (NAC) domain, and the C-terminal acidic domain. All identified disease-linked mutations are in the N-terminal domain. Mutation sites related to diseases are shown in red; these sites can be posttranslationally modified. **B** Transition of the monomeric form of α-syn to a pathological aggregate. Native forms of α-syn exist physiologically and can be folded into oligomers. Monomers form fibrils through a nucleated polymerization mechanism, and an equilibrium between fibrils and monomeric α-syn is maintained. The aggregation of amyloid fibrils results in the formation of intracellular deposits referred to as Lewy bodies (LBs)

The α-syn protein, encoded by the *SNCA* gene, is abundantly expressed in neuronal presynaptic terminals [34, 35]. Although its precise biological function is unclear, accumulating evidence indicates that α-syn plays a crucial role in regulating synaptic plasticity [36], synaptic vesicle release [37], molecular chaperoning [38], apoptosis [39], and oxidative stress [40], and it contributes to the pathogenesis of PD. α-Syn is a small 140-amino acid protein consisting of three distinct domains: the N-terminal domain (amino acids 1–65), the nonamyloid component of plaques (NAC) domain (amino acids 66–95), and the C-terminal domain (amino acids 96–140) [41–43] (Fig. 1a).

The N-terminus is a highly conserved domain with four copies of an imperfect 11-amino acid repeat that displays a KTKEGV consensus sequence; another three copies of this repeat are present in the NAC domain [44, 45]. This domain is amphiphilic, is unstructured in solution, and allows binding to phospholipid vesicles and the plasma membrane [46]. In addition, previous reports have indicated that several point mutations of in the N-terminus of α-syn are associated with autosomal dominant forms of familial PD [47]. The A53T and E46K mutations lead to a predisposition to α-syn fibril formation [48, 49], and the A30P mutation leads to a predisposition to the accumulation of α-syn oligomers [50, 51]. The NAC domain of α-syn, a central hydrophobic sequence, is essential for promoting α-syn aggregation [52], especially the NPC_61-95_ and NPC_71-82_ domains [53, 54]. A further study reported that the NAC domain's sixth and seventh 11-mer repeats can promote α-syn aggregation [55] and may serve as critical structures for acting on the β-amyloid (Aβ) protein to initiate the formation of amyloid plaques, a pathological feature of Alzheimer’s disease (AD) [56, 57]. The C-terminus of α-syn contains a high concentration of acidic amino acids [58] and 16 amino acid repeats essential for Ca^2+^ binding [59]. Compared to those exposed to full-length α-syn, neurons exposed to preformed fibrils (PFFs) containing residues 1–99 of α-syn exhibited a decrease in endogenous α-syn accumulation, indicating that the C-terminus of α-syn may facilitate aggregation [60]. C-terminal deletion mutants of α-syn without acidic repeats (residues 125–140) are vital for chaperone protein interactions and promote α-syn aggregation by eliminating chaperone activity [61, 62]. The phosphorylation and nitration of the second acidic repeat in the C-terminus can also disrupt the chaperone activity of α-syn [63, 64].

Several reports have shown that the conformational convergence of the α-helix with its β-sheet is essential for α-syn seeding and toxicity. Although oligomers with different β-sheet contents and degrees of hydrophobicity exist, they all possess a hollow cylindrical architecture with similarities with certain types of fibrils, suggesting that the β-sheet geometry is acquired in the early stages of oligomeric α-syn self-assembly [65], which generally requires approximately 30 monomers. However, the minimum number of α-syn monomers for stable fibril generation is approximately 70, which yields fibrils approximately 40 nm in size [66]. The existence of a bidirectional equilibrium between oligomers and higher molecular weight fibrillar assemblies suggests that cellular toxicity and seeding competency could be caused by different-sized conformers or assemblies [67] (Fig. 1b). Another important consideration is that conformational polymorphs of α-syn in distinct synucleinopathies and even specific cells spreading in different regions may support the spatiotemporal spread of α-syn within the brain [68].

## Heterogenous phenotypes of microglia responding to α-syn

Microglia are thought to maintain immune homeostasis in the CNS in response to various stimuli. Generally, activated microglia have been classically divided into two distinct phenotypes, the proinflammatory phenotype (M1 microglia) and the anti-inflammatory phenotype (M2 microglia) [69]. Rapidly emerging evidence suggests that microglia cannot be simply divided into two distinct categories; instead, they exist on a spectrum, with their phenotype changing along a continuum depending on the environment they inhabit [70–72]. Microglial phenotypic shift is related to neuroinflammation, as activated microglia are mainly responsible for the release of inflammatory cytokines, including IL-1β, IL-6, and TNFα [73, 74]. Lentivirus-mediated selective α-syn accumulation in microglia was used to investigate the transcriptome of model mice, and the results showed that the microglia in which α-syn accumulated exhibited heightened reactivity, characterized by phagocytic exhaustion and the presence of proinflammatory molecules [75]. Although α-syn is a potent activator of microglia, it is challenging to accurately assess microglial states throughout the various stages of PD due to the influence of the assessment method and timing of collection [70]. In addition, the complex and variable nature of microglial activation makes it challenging to determine activation status based solely on the stage and severity of neurodegeneration [76]. For example, monomeric α-syn-treated microglia exhibit significantly decreased iNOS expression and increased ARG-1 and CD206 expression levels, indicating the protective role of microglia under specific conditions. Microglia gradually transform into inflammatory microglia with prolonged α-syn incubation and increased iNOS and CD16/32 expression [77]. Recently, methodological advances in single-cell omics assays and integrative gene and protein expression analyses have enabled a deeper understanding of the molecular processes that underlie particular microglial activation states in a given context [78, 79]. Integrating published GWAS data with cell-type enrichment data, Reynolds et al. discovered that PD heritability through disease-relevant genes operates in a pathway-specific manner, with the lysosome-related gene set highly expressed in a microglial subpopulation [80]. To understand the involvement of glial cell types in PD and their impact on the severity of the disease, it is essential to utilize single-cell omics combined with advanced techniques and analysis tools in the future [81].

Highly polarized microglia are closely related to neuronal survival, and active microglia are related to disease type, disease stage, and even brain region [82–84]. The distribution of microglia is uneven across different brain areas, with the rat substantia nigra having abundant microglia [85]. Furthermore, evidence has suggested that microglia are proliferative and activated in the substantia nigra in PD patients [86] and animal models [87]. In addition to changes in synaptic function in the early stages, PD mouse models also exhibit differences in the number and location of microglia [88]. These findings suggest that microglia are responsible for the pathological process underlying dopamine neuron vulnerability and stage-dependent differences in PD progression.

## Involvement of α-syn in microglial pathology

**Fig. 2:**
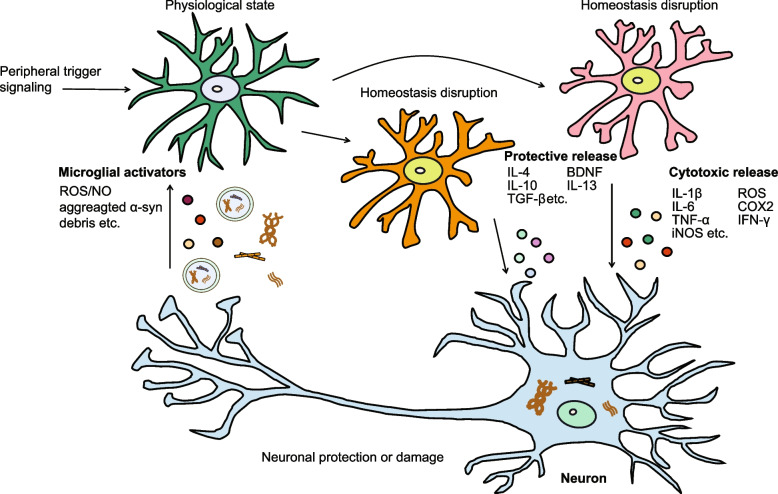
Schematic overview of the involvement of α-synucleinopathies and microglia in the pathogenesis of PD. α-Syn has the propensity to form polymeric and fibrillar structures, and the accumulation of α-syn in neurons can be toxic, resulting in neuronal degeneration and cell death. α-Syn in various conformations is released from neurons and recognized and internalized by microglia, leading to microglial homeostasis disruption. Microglia spatiotemporally release cytokines and cell survival factors, such as IL-4, IL-10, and BDNF*,* to restore the function of damaged neurons and the cytotoxic factors IL-1β, IL-6, and IFN-γ and substances such as reactive oxygen species (ROS), NO, and COX2 to promote α-syn aggregation and neuronal damage

Experimental and clinical studies have provided evidence that α-syn propagation contributes to the anatomical spread of Lewy pathology. Although α-syn is typically considered an intracellular protein, several studies have identified its presence in the cerebrospinal fluid (CSF) and plasma of individuals with PD [89, 90]. It has been observed that primary cortical neurons from rats and neuron cell lines can secrete extracellular vesicles containing α-syn [91]. Furthermore, when intracellular protein transport through lysosomes is blocked, there is an increase in the release of extracellular vesicles containing α-syn, suggesting that modulating intracellular α-syn processing may significantly contribute to its release [92–94]. Accumulating research has highlighted the crucial role of microglia in transmitting harmful protein aggregates between cells, which is thought to contribute to the spreading of α-syn neuropathology and underlie the pathogenesis of PD [95]. Microglia have a strong capacity to take up surrounding α-syn containing exosomes, leading to their activation and subsequent intracellular processing [96, 97]. Blocking exosome activation in microglia has been found to decrease the spread of α-syn to neurons [98]. In pharmacological ablation experiments targeting host microglia in the striatum, it was observed that α-syn accumulation was increased in α-syn-expressing dopaminergic neurons, and α-syn was transferred to neurons via a process modulated by microglial inflammatory activation [99]. These studies provide insight into the noncell-autonomous effect associated with the intracellular accumulation of α-syn and its interneuronal transmission, indicating that microglia are functionally involved in the disease pathogenesis (Fig. 2).

### Pathways by which microglia internalize α-syn

The internalization of α-syn by resting and activated microglia is a critical step that drives neuroinflammatory responses, and multiple processes underlying this function have been proposed. Endocytosis is thought to be the primary process by which pathological proteins are internalized. Endocytosis can be divided into two categories: clathrin-independent and clathrin-dependent endocytosis. Particles that are larger than 0.5 μm can be internalized by clathrin-independent endocytic mechanisms, such as micropinocytosis and phagocytosis [100], and particles that are smaller than 200 nm can be internalized through dynamin-dependent or dynamin-independent pathways [101].

**Fig. 3:**
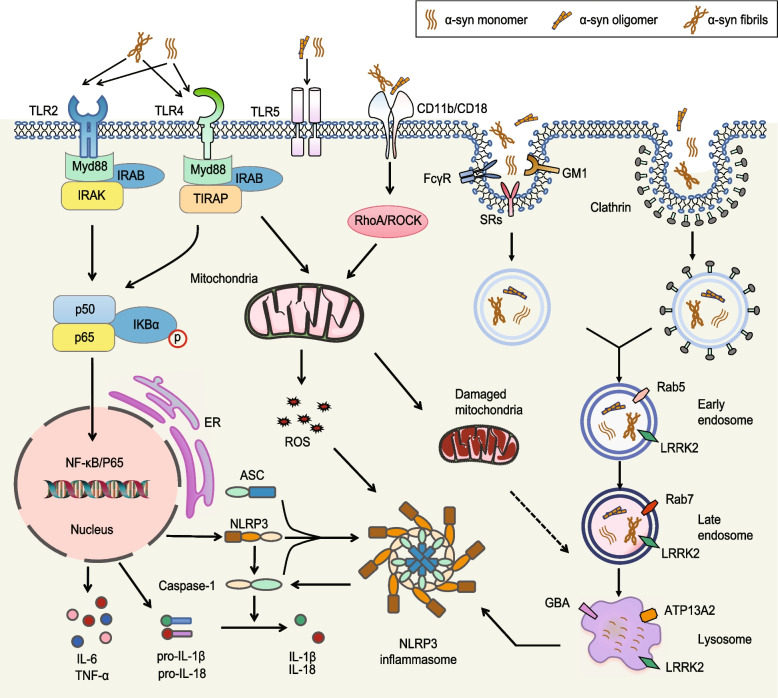
Involvement of α-syn in microglia-mediated cellular processes in PD. Microglia recognize and internalize monomeric, oligomeric, and fibrillary α-syn through cell surface receptors, including the Toll-like receptors (TLRs) 1/2, 4, and 5, and intracellular proinflammatory pathways are subsequently activated via the transcription factor NF-κB/P65. Additionally, α-syn upregulates NLRP3 (NOD, LRR-and pyrin domain-containing 3), which results in the assembly of apoptosis-associated speck-like protein containing a CARD (ASC) into specks and activation of caspase 1 to form the inflammasome. Alternatively, the binding of different forms of α-syn to TLR4 or CD11b induces mitochondrial dysfunction and facilitates the release of mtROS, thus inducing the assembly and activation of the NLRP3 inflammasome. α-Syn internalization triggers endocytosis as well as both clathrin-dependent and clathrin-independent processes. PD-related genes, such as *LRRK2*, regulate the stages of endocytosis-lysosomal assembly in microglia and might contribute to inflammation and neuronal α-syn pathology

Microglial phagocytosis is a process by which microglia can recognize and take up extracellular targets, including abnormally aggregated proteins, via specific receptors [102]. Compared with that of wild-type microglia, the phagocytic capability of microglia from *SNCA*^*−/−*^ mice was found to be reduced, suggesting that endogenous α-syn might exert a positive effect on the phagocytic ability of microglia [103]. Monomeric α-syn, but not β- or γ-syn, was observed to boost microglial phagocytosis in a dose- and time-dependent manner. Several receptors are critical for activating microglial phagocytosis and the ability of microglia to remove α-syn [29, 104, 105]. Microglial TLR4 deficiency was found to diminish α-syn phagocytic activity. TLR-4 mediates microglial phagocytosis of full-length soluble α-syn, fibrillary α-syn, and C-terminally truncated α-syn, and the latter has been identified as have more potent effects than the other forms [106]. Nevertheless, the uptake of α-syn by astrocytes with TLR4 deficiency did not abolish phagocytosis, indicating a potential cell type-specific difference in the uptake of α-syn. The expression of CD22, a negative regulator of microglial homeostasis, is significantly elevated in microglia-conditioned α-syn-expressing CX3CR1-SNCA mice, and CD22 significantly abolishes microglial phagocytosis [75, 107]. According to one study, monomeric α-syn can enhance microglial phagocytosis, while aggregated α-syn can suppress this effect [29], suggesting that microglia show different responses when they phagocytose distinct forms of α-syn. A further investigation discovered that the aggregated form of α-syn directly binds to and facilitates FcγRIIB expression, resulting in the activation of SHP-1, a suppressor of microglial phagocytosis [108], supporting the observation that highly complex form of α-syn may attenuate microglial phagocytosis. An in vitro study demonstrated that immortalized BV2 microglia-like cells take up α-syn via ganglioside GM1 by linking to lipid rafts at the plasma membrane [109] or directly binding to macrophage antigen-1 (Mac-1) receptor [110] (Fig. 3).

It has been proposed that several cell-surface receptors, including TLR2 and TLR4, are involved in α-syn internalization by clathrin-dependent endocytosis [111, 112]. The uptake of α-syn by clathrin-mediated endocytosis has been demonstrated in primary cultured microglia [113]. However, blocking this pathway cannot fully inhibit α-syn uptake, suggesting that alternative routes are involved in α-syn internalization [114]. It was also found that soluble monomeric α-syn directly translocates across the plasma membrane in a manner not involving endocytic receptors [114, 115].

### Intracellular processing of internalized α-syn

Although α-syn aggregates have been observed to be internalized and cleared by microglia, the precise intracellular events responsible for this process is still not fully understood. Studies of familial PD mutation genes have demonstrated that the internalization of α-syn into recipient cells typically leads to its degradation by lysosome vesicles. For example, PD-associated genes carrying causal mutations, such as the *LRRK2* mutation identified from patients with autosomal-dominant monogenic familial cases of PD, have been reported to be associated with lysosome processing of α-syn within microglia (Fig. 3). LRRK2 is present on lysosomes [116] and plays a direct role in endocytosis through its interactions with Rab small GTPases, which regulate the fusion of certain endosomes and consequent lysosomal content degradation [117] and synaptic vesicle recycling [118]. Microglial knockout of LRRK2 increases the amount of α-syn taken up and the α-syn clearance efficiency, which is correlated with an increase in the number of Rab5-positive endosomes [31]. Interestingly, LRRK2 has been reported to play a positive role in microglial phagocytosis of neuronal axons and elements [116]. This suggests that LRRK2 strongly controls vesicular trafficking events within endolysosomes and has a wide-ranging influence on endocytosis and the degradation of unfolded/aggregated proteins in microglia. However, the influence of PD-related genes, such as ATP13A2, its common risk variants and GBA, which are linked to lysosomal activity in neurons and astrocytes [112], on the endolysosomal system in microglia is yet to be determined, and thus, further investigation is needed. Another study suggested that after a short incubation time, α-syn selectively induces the expression of p62/SQSTM1 [119], an autophagy-related protein that recognizes ubiquitinated proteins and interacts with these proteins to mediate selective autophagy for clearance [120], through the TLR4/NF-κB pathway. Knocking out p62/SQSTM1 prevents the effective removal of α-syn and aggravates pathology [119].

### Signaling pathways involved in α-syn-induced microglial inflammation

#### Activation of the NF-κB signaling pathway by α-syn

In α-syn-related pathology, the expression level of the microglial surface receptor TLR2 is significantly increased. Oligomeric or fibrillary α-syn activates the microglia surface receptor TLR2, causing intracellular NF-κB pathway activation and the release of inflammatory factors such as TNF-α and IL-6 [111, 121]. The regulation of downstream signals by TLR2 requires its adaptive protein MyD88 [122]. Suppressing the effect of MyD88 on TLR2 can effectively inhibit the activation of NF-κB signaling and alleviate α-syn-induced neuroinflammation and dopaminergic neuron loss [24]. TLR4 regulates microglial phagocytosis of α-syn, activates the intracellular NF-κB signaling pathway, and causes the release of inflammatory factors [123]. The various forms of α-syn have distinct preferences for TLR2 and TLR4, with their effects on these receptors varying depending on the form of α-syn. At low concentrations, monomeric α-syn preferentially acts on TLR4 to induce selective autophagy to clear α-syn, which may be related to its ability to regulate microglial phagocytic activity [124]. In contrast, oligomeric or fibrillary α-syn-activated microglia induce inflammation through cell-surface TLR2. Studies have shown that the α-syn promoter contains NF-κB binding sites, so α-syn-induced NF-κB activation further causes α-syn expression and aggravates α-syn-induced pathology, forming a vicious cycle [125]. A study investigated the consequences of exposure to wild-type α-syn and three α-syn mutants (A53T, A20P, and E46K) on microglial reactivity. It was found that α-syn-induced microglial reactivity appeared to be peptide dependent, and the inflammation caused by the A53T mutant was more substantial than that caused by the wild-type form [126]. Although short-term exposure to the A53T mutant may stimulate MAPK-pERK pathway activation, the long-term inflammatory response elicited by continued exposure to the mutant protein requires further investigation.

#### α-Syn affects inflammasome formation in microglia

Some reports have suggested the importance of the NLR family pyrin domain containing 3 (NLRP3) inflammasome, a persistent source of microglia-mediated inflammation that drives progressive dopaminergic neuropathology in PD [127, 128]. Activation of the NF-κB pathway promotes NLRP3 inflammasome assembly and IL-1β release and further causes mitochondrial dysfunction, reactive oxygen species (ROS) production, and oxidative stress [129]. RNA-sequencing (RNA-seq) analysis of the substantia nigra of 5-week-old *CX3CR1-SNCA* mice showed that α-syn aggregation in microglia significantly increased the expression of inflammation-related genes, including the inflammasome components NLRP3 and adaptor protein apoptosis-associated speck-like protein containing a CARD (ASC), IL-1β, and NF-κB [75]. This observation indicates that α-syn in microglia activates the NLRP3 inflammasome and induces the release of IL-18 and IL-1β, consistent with previous in vitro studies. Inhibition of NLRP3 inflammasome activity significantly alleviates PD pathology, including neuroinflammation and motor dysfunction [127, 128]. In chronic MPTP-treated NLRP3 knockout mice, NLRP3 deletion was observed to inhibit inflammasome formation and protect against α-synuclein pathology by alleviating autophagy dysfunction [130]. α-Syn-mediated activation of the inflammasome component NLRP3 in microglia promotes ASC release and induces α-syn aggregation in neurons [75].

Research has revealed that the assembly of the NLRP3 inflammasome requires two distinct steps: priming and activation. According to research, α-syn fibrils can initiate intracellular NLRP3 inflammasome assembly or activate the NLRP3 inflammasome through cell surface receptors [131, 132]. These molecules act as priming signals to activate the NLRP3 inflammasome. For instance, α-syn fibrils interact with TLR2, activating downstream signals to activate the NF-κB pathway, which, in turn, upregulates NLRP3, ASC, and pro-caspase-1 [133], and anti-TLR2 antibodies attenuate this response. Scheiblich et al. reported that α-syn monomers and oligomers, but not α-syn fibrils or ribbons, activate the NLRP3 inflammasome via TLR2 and TLR5 under unprimed conditions [134]. In addition, microglial TLR2 and TLR5 neutralization by specific antibodies blocks caspase-1 cleavage and NLRP3 inflammasome activation induced by α-syn monomers and oligomers. These observations indicate that α-syn monomers acting on TLR2 coordinate with α-syn monomers and oligomers acting on TLR5 to contribute to the priming step of NLRP3 inflammasome activation. Another study suggested that α-syn interacting with TLR4 promotes the expression of proinflammatory cytokines and increases mitochondrial ROS (mtROS) levels by damaging mitochondria and triggering NLRP3 inflammasome assembly and activation [135]. An additional study suggested that α-syn fibrils can activate the NLRP3 inflammasome under unprimed conditions; however, it was found that the expression level of IL-1β is lower in the unprimed state than in the primed state but significantly higher than that in untreated microglia [136]. These conflicting results may be due to differences in the concentration and/or quality of the prepared α-syn aggregates and may indicate the existence of alternative pathways involved in NLRP3 inflammasome activation.

For instance, α-syn can bind to CD11b to initiate NLRP3 inflammasome assembly. CD11b is highly expressed on microglia in the CNS and has been reported to regulate microglial phagocytosis and migration [137, 138]. α-Syn fibrils bind to CD11b to cause mtROS production through the RhoA/ROCK pathway to activate the NLRP3 inflammasome [139]. The interaction between oligomeric α-syn and CD11b induces NOX2 activation and the production of hydrogen peroxide, which binds to an intracellular tyrosine-protein kinase known as Lyn, resulting in actin phosphorylation and cytoskeleton rearrangement [140]. This ultimately leads directly to the induction of microglial migration to damaged nerve cells, which are cleared through phagocytosis [141]. Microglial activation may lead to neuronal repair; however, persistent activation of microglia for an extended period may cause indiscriminate phagocytosis of neuronal synapses, disrupting neurotransmission.

### Link between α-syn accumulation and microglial inflammation

Some studies suggest that aggregated α-syn promotes M1 microglial polarization [30, 142] and causes monomeric α-syn aggregation, implying that the activation of the microglial immune response might be intrinsically linked to the accumulation of α-syn [131]. Cell-surface Toll-like receptors (TLRs) appear to be key molecules in the signaling pathway that link α-syn phagocytosis with the formation and transport of phagosome cargos into lysosomes for degradation [124, 143]. Evidence has demonstrated that TLR1 [144], TLR2 [145], TLR4 [106], and TLR5 [134], members of the TLR family, functionally interact with α-Syn, with TLR2 and TLR4 playing a more prominent role. Primary microglia exhibit significantly increased expression of TLR1 and TLR2 after exposure to mutant A53T α-syn [145]. Studies indicate that TLR2 can recognize monomeric, oligomeric, and fibrillar forms of α-syn and trigger the intracellular cascade to promote inflammatory activation [125, 146]. Evidence suggests that TLR4 is highly expressed in patients with synucleinopathies and is vital for the uptake of α-syn and the associated inflammatory processes, including the generation of ROS and cytokine release [106]. Upon TLR activation [106], the NF-κB cascade is triggered, leading to the transcription of p62, an autophagy receptor essential for forming a platform for autophagosome formation to eliminate ubiquitinated proteins in microglia [147].

Myeloid cell-triggered receptor II (TREM2), a member of the immunoglobulin superfamily, is abundantly expressed on the surface of microglia in the CNS and plays a critical role in microglial phagocytosis, proliferation, and inflammatory factor production [148, 149]. Studies have revealed that TREM2 is a contributing factor to PD development [150], and genetic research has revealed that the R47H mutation (rs75932628, p.R47H) in TREM2 contributes to the development of both PD and AD [151]. Studies on mouse models of PD induced by MPTP have revealed that TREM2 can reduce pathological changes in the nervous system and inhibit NF-κB, MAPK, and TLR4/TRAF6 signaling during the neuroinflammatory response [152]. Research has demonstrated that overexpressing TREM2 inhibits the neuroinflammatoty response through the PI3K/AKT and NF-κB signaling pathways in LPS-stimulated BV2 cells [153]. Transfection of BV2 microglia with TREM2-siRNA exacerbated the inflammatory responses induced by α-syn and conditioned medium, triggering apoptosis in cultured SH-SY5Y cells [154]. These observations have been confirmed in TREM2 knockout mice with AAV-mediated α-syn overexpression, as it was found that dopaminergic neuron loss is exacerbated in these mice [155]. One clinical study showed that the levels of TREM2 in the CSF of PD patients were elevated compared to those in the CSF of healthy controls and had a positive correlation with total α-syn degradation [156]. These findings demonstrate the potential bidirectional role of TREM2 in processing α-syn on the cell membrane, as well as in inducing microglial inflammation.

The aromatic residues of α-syn can form hydrogen bonds with glycosphingolipids in the cell membrane, forming a round porous oligomeric structure, causing intracellular calcium current disorder, interfering with intracellular homeostasis, and leading to cell degeneration [157]. Oligomers have a higher membrane-binding capacity than monomers, and their binding to lipids is considered the early stage in the development of synucleinopathies [158]. In addition, α-syn aggregation can induce microglia to release the neurotransmitter glutamate, resulting in extracellular glutamate accumulation and CNS excitotoxicity [159]. Metabotropic glutamate receptor 5 (mGluR5) is a G-protein coupled receptor (GPCR) present in neurons and microglia. Stimulating mGluR5 in microglia is a desirable strategy, as it can significantly inhibit the inflammatory response; thus, mGluR5 may play a critical role in microglial homeostasis [160, 161]. mGluR5 agonists can inhibit α-syn-induced inflammatory signals and cytokine secretion in BV2 cells and primary microglia and reduce microglial activation to protect against neurotoxicity. Additionally, mGluR5 was found to colocalize with α-syn in lysosomes. Studies of an α-syn-based PD rat model revealed that α-syn promotes the breakdown of lysosome-dependent degradation of the mGluR5 complex to control neuroinflammation [161].

### Microglia mediate α-syn release and transmission between cells

The release of α-syn is associated with exosomes, as exosomes containing α-syn are formed through the fusion of multivesicular bodies with the plasma membrane, a process that is affected by the intracellular Ca^2+^ concentration [162–164]. Upon treatment with α-syn preformed fibrils, exosomes containing α-syn released by donor microglia can induce protein aggregation in recipient neurons [97]. Exosomes containing pathological α-syn may also be internalized by neighboring microglia [165] or other cells by receptor-mediated endocytosis [166]. In an in vitro culture system, it was found that LPS promoted the secretion of soluble factors and TNF-α from activated microglia and increased α-syn levels in the medium. Importantly, TNF-α secreted from LPS-treated microglia promotes cell-to-cell propagation of α-syn between neurons, while soluble factors trigger cellular senescence. Interestingly, the secretion of α-syn was found to be increased in senescent neurons, indicating the acquisition of a senescence-associated secretory phenotype (SASP) in response to TNF-α stimulation. Related morphological studies have confirmed that propagating α-syn aggregates are present in electron-dense lysosome-like compartments, indicating that inflammatory factors can drive cell-to-cell propagation of α-syn by promoting the acquisition of a SASP and the secretion of α-syn [167]. Recently, studies have suggested that microglia can form tunneling nanotubes for microglia-to-microglia spread of α-syn [168]. In addition, α-syn-induced activated microglia promote the release of α-syn-containing exosomes and increase α-syn propagation [97, 166]. The pathways through which α-syn is secreted by microglia may serve as essential mechanisms for amplifying and spreading α-syn pathology to the surrounding milieu.

Recently, lymphocyte activation gene 3 (LAG3) was shown to act as a receptor for pathogenic α-synuclein assemblies in the CNS in PD [169]. Further investigation found that the C-terminus of α-syn binds to LAG3 and amyloid precursor-like protein 1 (APLP1)-positive pockets, facilitating the entry of α-syn into neurons [170]. The phosphorylation of α-syn at S129 in the C-terminus promotes α-syn fibril binding to LAG3 and APLP1, enhancing the propagation of α-syn fibers in neurons [171]. Overexpression of LAG3 increases the phosphorylation of α-syn, thus exacerbating PD pathology, whereas deletion of LAG3 has the opposite effect, inhibiting α-syn neuronal transmission, PFF-induced neuron loss and toxicity both in vitro and in vivo [169]. Increasing evidence suggests that LAG3 may be involved in the endocytosis and intercellular transmission of α-syn [172, 173]. In mouse cortical neurons, deletion of LAG3 can recruit α-syn to early endosomes, where it colocalizes with RAB5, significantly reducing α-syn aggregation; this indicates that LAG3 is involved in processing α-syn during endocytosis [169, 174]. snRNAseq and scRNAseq data suggest that microglia express LAG3, and the administration of an anti-LAG3 monoclonal antibody was found to protect against neuronal impairment [175], indicating a modulatory role for LAG3 in α-syn pathology through microglial pathways.

## Peripheral interference with microglial inflammation and α-synucleinopathy

Multiple clinical and preclinical studies have provided evidence that PD pathology can be transmitted from the peripheral to central nervous systems and trigger neuroinflammation through various mechanisms, including the regulation of the gut microbiota and disruption/disturbance of ascending or descending neurotransmission. A study revealed the existence of a bidirectional connection between the CNS and the gastrointestinal system, known as the "brain-gut axis", despite their anatomical separation. Intestinal bacterial colonization regulates immune system maturation and development in the CNS. It has been reported that α-syn-dependent microglial activation may be linked to changes in the microbiome in both animal models and patients [176]. According to these observations, the gut microbiota is involved in the pathogenesis of PD, and its role is mediated by bidirectional regulation of the immune response of microglia in the CNS.

A series of well-designed experiments demonstrated that α-syn-overexpressing mice harboring a complex microbiota have worse motor function and constipation, accompanied by marked microglial neuroinflammatory responses and α-syn aggregation in the brain, than α-syn-overexpressing germ-free (GF) mice (born and raised in sterile environments). Gut microbiota transplantation from PD patients into GF α-syn-overexpressing mice was found to exacerbate motor dysfunction [177]. Intriguingly, using antibiotics to eliminate the microbiota in PD model mice was shown to alleviate motor dysfunction and pathological features [176]. Increasing the gut microbiota diversity in PD model mice improves motor function and ameliorates pathological features [176]. GF mice display global microglial defects, such as altered cell proportions, defects in microglial maturation, and impaired innate immune responses. However, another critical study highlighted the substantial contribution of host-dwelling bacteria to microglial homeostasis. In GF mice, microglia in the brain are globally defective, exhibiting altered proportions and maturation. The expression of the microglial surface molecules CSF1R, F4/80, and CD31, which gradually decreases during cell maturation in the brain, was found to be increased. These findings indicate that host bacteria are vital for microglial development and function, and that microglial impairment can be at least partially rectified by a complex microbiota [178, 179]. Gut microbiota supplementation, especially with short-chain fatty acids (SCFAs) and microbial metabolites, promotes the maturation of microglia in GF mice, suggesting that the gut microbiota regulates the development and growth of microglia. Indeed, intensive studies have shown the potential effects of the gut microbiota on the pathogenic features of PD in various models, including *Drosophila melanogaster* [180, 181], mouse [176, 182, 183] and nonhuman primate [184, 185] models. Additionally, clinical studies have shown gut microbiota changes in PD patients [186, 187]. *Akkermansia*, *Bifidobacterium,* and *Lactobacillus*, bacteria commonly considered beneficial, are increased in abundance in PD [188], but the mechanisms underlying this change are unknown.

Accumulating evidence indicates that inflammation caused by gut dysbiosis involves α-syn transmission from the gut to the brain and/or α-syn aggregation during PD progression. The transmission of α-syn from the gut to the brain, accompanied by the degeneration of dopaminergic neurons in the substantia nigra and PD-like motor and nonmotor symptoms, has been shown to occur in rodent models [182, 183]. Aged rats receiving gastrointestinal injections of aggregated α-syn show more efficient gut-to-brain spreading than young rats, suggesting that aging is a critical risk factor for α-syn transmission via this pathway [189, 190]. Studies have shown that there are alterations in gut microbiome transmission and the effects of microglia-related neuroinflammation within the brain, and studies on intestinal microbiome perturbations have also shown the relationship between intestinal hyperinflammation and local α-syn aggregation. For example, marmosets with colitis show increased phosphorylated α-syn accumulation in the colonic myenteric plexus [185]. In addition, transgenic mice overexpressing α-syn show gut microbiota alterations [191]. Therefore, more studies are needed to clarify the molecular signaling pathways involved in the influence of the microbiota on microglial activation.

Various potential pathways, such as bidirectional endocrine and immune pathways involving the brain-gut-microbiota axis, might be involved in the pathological processes of PD [192]. The blood‒brain barrier (BBB) can prevent harmful substances from entering the brain through the blood, and it plays an essential role in maintaining the normal physiological state of the CNS and preventing pathogens from invading the brain [193]. PD affects the permeability of the BBB and causes abnormally deposited proteins, such as α-syn, in the CNS to diffuse into the intestine through the BBB, affecting the gut microbiota balance. Nevertheless, studies have suggested that gut microbiota impairment affects the permeability of the BBB, thus allowing intestinal microbial metabolites and intestinal inflammatory factors to enter the CNS [194]. In addition to microglia, peripheral immune cells and activators can cross the BBB under pathological conditions [193]. For instance, microglia-specific expression of α-syn induces the release of several chemokines, such as CCL1, CCL2, and CXCL10 [75]. These chemokines increase inflammation and recruit peripheral immune cells, including CD4^+^ and CD8^+^ T cells, to the brain, thus exacerbating inflammation [75, 195]. T helper 17 (Th17) cells, a subtype of CD4^+^ T cells, enter the brain through the CXCR4-CXCL12 pathway and interact with microglia to interact with α-syn and participate in IL-17A-regulated neuronal injury [196]. Furthermore, GF mice show increased BBB permeability [197], and BBB permeability can be decreased by SCFA-producing bacteria and oral gavage with solidum butyrate [198].

Evidence of some important neuroanatomical pathways in PD has led to the proposal of the "dual-hit" hypothesis to explain the disease's peripheral onset [199]. Hyposmia and constipation are common nonmotor symptoms of PD that can appear years, or even decades, before diagnosis, and Lewy-type pathology can be detected in peripheral tissues up to twenty years before diagnosis. Truncal vagotomy disrupts α-syn transmission from the gut to the brain in animals [183] and substantially reduces PD risk in patients [200]. Animal and clinical studies have demonstrated that LB-like aggregates are deposited in the enteric nervous system (ENS) and dorsal motor nucleus of the vagus nerve (DMV) at an early stage and are linked to the severity of motor and gastrointestinal symptoms [201, 202]. Consistent with these findings, injection of α-syn into intestinal tissues can induce α-syn pathology in the vagus nerve and brainstem [182]. However, this evidence has not been confirmed clinically, as there is a lack of postmortem patients with isolated gastrointestinal α-syn pathology without accompanying CNS pathology. Despite the controversy concerning the peripheral origin of diseases, this theory provides an intriguing perspective and significant directions for the development of treatments for PD.

## Therapeutic strategies targeting α-syn aggregation and neuroinflammation

α-Syn aggregation and transmission and neuroinflammation play crucial roles in the pathogenesis of PD, and unsurprisingly, reducing α-syn aggregation and inhibiting inflammation have become fundamental goals of PD therapy. To this end, clinical and preclinical trials of approaches for preventing α-syn aggregation and inflammation have been carried out or are underway.

The ability of various approaches targeting different points in the life cycle of α-syn to prevent or lessen its harmful effects in PD is being investigated. Immunotherapies targeting pathological α-syn are currently being developed as primary therapeutic approaches and tested in clinical trials [203]. In addition to immunotherapies, including those that suppress α-syn (NTP200-11) [204], reduce α-syn transcription [205, 206] or translation [207], eliminate pathological aggregates [208–211], and enhance α-syn clearance and degradation [212–214], have been tested as disease-modifying treatments for α-synucleinopathies. In addition to blocking the release of α-syn from donor cells in the CNS, preventing α-syn transmission from the peripheral nervous system has recently been identified as an effective therapeutic strategy [183]. An alternative approach is to target other genes or proteins associated with or implicated in PD that contribute to the processing or aggregation of α-syn, such as Gcase, LRRK2, NMDAR, and AMPAP [215–218]. The proteolytic system is an efficient mechanism for eliminating abnormally deposited proteins within cells, and regulating protease expression and enzymatic activity is important for facilitating α-syn clearance [219]. For example, the expression of the protease cathepsin D, which is a component of the proteolytic system, is reduced during α-syn aggregation, and this protease protects against α-syn-induced cell death in vitro and in *C. elegans* models [220]. These findings suggest that the proteolytic system efficiently eliminates abnormally accumulated proteins within cells, highlighting its potential as a crucial target for PD treatment.

Targeting inflammation has emerged as a potential therapeutic approach for PD, as it may help to slow or halt the progression of the disease. Inflammation damages nerve cells and promotes the expression of α-syn, further aggravating PD pathology [125]. Studies have highlighted innate and adaptive immune response abnormalities in PD patients, including increased proinflammatory cytokine levels and altered immune cell populations such as monocytes and their precursors [221]. Neuroinflammatory features have been observed in clinical studies and experimental models of PD, thus establishing a link between inflammation and the pathogenesis of this condition [222, 223]. TLR plays a crucial role in activating microglia-induced neuroinflammation mediated by α-syn. Treatment with an anti-TLR2 antibody was shown to significantly decrease α-syn deposition and neuroinflammation in animal models of PD, leading to alleviation of movement disorders [224]. Additionally, CU-CPT22, a small molecule inhibitor targeting TLR1/2, attenuates oligomeric α-syn-induced inflammation in primary microglia [121]. Upregulation of NLRP3 in microglia has been demonstrated to significantly upregulate inflammatory cytokines, transcription factors, and critical components of the inflammasome pathway, contributing to dopaminergic cell loss in PD mouse models [225]. MCC950, a small molecule inhibitor targeting the NLRP3 inflammasome, has been demonstrated to suppress neuronal damage, attenuate α-syn deposition and inflammation, and alleviate dyskinesia in various PD models, including PFF- and 6-OHDA-treated and α-syn A53T mutant animals [127, 128].

Autoantigens, such as pathological α-syn and inflammatory factors, which can be presented on the surface or secreted into the extracellular environment by neurons, activated microglia, and macrophages in the brain and CSF [226, 227], can increase BBB permeability and thus allow T-cell entry, resulting in detrimental effects on neurons [221, 228, 229]. Sargramostim, an FDA-approved recombinant form of human granulocyte–macrophage colony-stimulating factor (GM-CSF), has been demonstrated to enhance regulatory T-cell function and improve motor outcomes. Due to its antineuroinflammatory properties, it is currently being investigated as a potential therapeutic option for PD [230]. Epidemiological evidence suggests that the secretion of TNF-α by activated microglia in patients is associated with an elevated risk of developing PD [231], while anti-TNF therapy has been linked to a significant reduction in PD incidence and promotes the survival of dopaminergic neurons [232, 233]. Consistent with these observations, anti-TNF antibodies prevent the death of dopaminergic neurons in mice [234, 235]. The transcription factor PPARγ, expressed in neurons and glia, is a molecular link between glucose metabolism and the regulation of microglial inflammation [236]. The PPARγ agonist pioglitazone has been used in the clinic for T2DM treatment, and its effectiveness was recently evaluated in PD animal models [237]. The PPARγ agonist rosiglitazone effectively inhibits LPS-induced microglial activation, whereas the antagonist T0070907 induces a shift of microglia from an inflammatory phenotype to a homeostasis-restoring phenotype [238]. The PPARγ agonist exerts inhibitory effects on microglial activation, leading to a reduction in the production of proinflammatory factors and protecting dopaminergic neurons by modulating multiple signaling pathways, including the JUN, NK-κb, and NF-AT pathways.

The involvement of the gut microbiota in chronic inflammation and α-synuclein aggregation in the enteric nervous system presents new therapeutic opportunities that are largely unexplored. Notably, an ongoing clinical trial (NCT03958708) is investigating the effects of rifaximin, an antibiotic, in reducing systemic inflammation and α-synuclein aggregation by targeting the gut microbiota in individuals with PD. Another study (NCT03808389) is exploring the potential benefits of fecal transplantation in alleviating gut inflammation in PD patients. While most of these trials are primarily assessing clinical motor endpoints or the ability of the treatments to act on their targets, evaluating immune-related endpoints and outcomes is crucial in preclinical and clinical studies owing to the close relationship between synucleinopathies and neuroinflammation.

## Conclusions, limitations, and perspectives

This review emphasizes the importance of microglia, which are located in the inflammatory environment within the CNS, in establishing a connection between neuroinflammation and α-synucleinopathies associated with PD. The exact mechanism by which disruption of microglial homeostasis contributes to α-synucleinopathy is still under investigation, but several findings suggest that microglia may act as regulators of this process. Despite the wealth of information presented in this review regarding microglia-related pathological changes, there remains significant uncertainty surrounding the specific states of microglia and their role in the pathogenesis of PD. Additionally, recent research has highlighted distinct functional variations among microglial phenotypes across different brain regions, potentially contributing to the unique patterns of microglial-mediated inflammation in PD [239]. The complexity of cellular phenotypes extends beyond conventional classifications, suggesting that further investigation is imperative for elucidating inflammation signatures associated with neurodegenerative diseases [240]. Therefore, developing enhanced methods such as scRNA-seq or spatial transcriptomics for characterizing microglial signatures in humans or disease models would be highly advantageous to the field.

Several large-scale epidemiological and clinical studies have provided limited evidence of a relationship between intestinal diseases, gut-targeted interventions, microbiome changes, microglial homeostasis, and α-synucleinopathies [241]. Inflammation in the gut contributes to disease development through systemic mechanisms such as increased cytokine production, disruption of the blood brain barrier, migration of inflammatory cells into the brain, and activation of microglia according to studies of PD patient biopsies and fecal samples. Identifying gut microbes and metabolites that cause the disease is extremely challenging [242], as they can act independently or exert enhancing or counteracting effects within the microbial community. However, recent advances in technical and computational tools used to investigate the composition and function of the microbiome could facilitate the analysis of variations in the influence of host-associated microbial communities [243] and may provide clues for understanding the communication between the microbiome and microglia in the progression of α-synucleinopathies. 

The current understanding of microglial states and their involvement in α-synucleinopathies is derived from studies utilizing diverse animal models, cell culture systems, and human samples. Researchers have made significant progress in the field by reprogramming primary microglia from fresh postmortem brains of individuals with disease or stem cells obtained from human or animal models to become microglia-like cells, enabling access to more rapid and physiological findings [244]. Recent advances in the use of human induced pluripotent stem cell (iPSC)-derived microglia-like cells (iMGLs) have allowed successful recapitulation of disease phenotypes, providing a better understanding of the pathological roles of microglia in neurological diseases [245, 246]. Alternatively, the monocyte-derived microglia-like cell (MDMi) model is another in vitro culture system that  both recapitulates the genetic background of the humans from which the cells are derived [247] and allows for rapid large-scale cultivation. This system may be beneficial for exploring the interaction between the disruption of microglial homeostasis and disease progression [248, 249]. When investigating the disruption of microglial homeostasis and α-syn, model selection should be contingent on the context, with the model cells being cultured either alone or in combination with other cells, to obtain the most robust findings that reveal pertinent disease pathways. Furthermore, these findings should be cross-validated in other systems according to downstream applications to evaluate potential treatment methods.

In summary, microglia play a pivotal role in central inflammation, and the interaction of these cells with α-syn may contribute to the development of PD pathogenesis; thus, microglia are indispensable targets for therapeutic interventions.

## Data Availability

Not applicable.

## Abbreviations

LBs: Lewy bodies
LNs: Lewy neurites
α-syn: α-synuclein
PD: Parkinson's disease
SNpc: Substantia nigra pars compacta
GWAS: Genome-wide association study
DLB: Dementia with Lewy bodies
MSA: Multiple system atrophy
LPS: Lipopolysaccharide
CNS: Central nervous system
NAC: Nonamyloid component of plaques
TNF-α: Tumor necrosis factor α
IL-1β: Interleukin 1
IL-6: Interleukin 6
MHC: Major histocompatibility complex
IL-4: Interleukin 4
IL-10: Interleukin 10
ARG-1: Arginase-1
mGluR5: Metabotropic glutamate receptor 5
GPCR: G-protein coupled receptor
KLK6: Kallikrein
EVs: Extracellular vesicles
PTMs: Posttranslational modifications
L-DOPA: Levodopa
LAG3: Lymphocyte activation gene 3
APLP1: Amyloid precursor-like protein 1
NF-κB: Nuclear factor kappa-B
TLR4: Toll-like receptor 4
CXCL1: Chemokine ligand 1
iNOS: Inducible nitric oxide synthase
COX-2: Cyclooxygenase-2
TLR2: Toll-like receptor 2
Myd88: Myeloid differentiation factor 88
NLRP3: NLR family pyrin domain containing 3
ROS: Reactive oxygen species
ASC: Adaptor protein apoptosis-associated speck-like protein containing a CARD
H_2_O_2_: Hydrogen peroxide
BBB: Blood‒brain barrier
GF: Germ-free
LCMV: Lymphocytic choriomeningitis virus
SCFAs: Short-chain fatty acids
NMDAR: N-methyl-D-aspartate receptor
AMPAP: α-Amino-3-hydroxy-5-methyl-4-isoxazolepropionic acid receptor
TSPO: Translocator protein
GLP1: Glucagon-like peptide 1
T2DM: Type 2 diabetes mellitus
iMGLs: IPSC-derived microglia-like cells
MDMi: Monocyte-derived microglia-like cells
